# Imprecise Predictive Coding Is at the Core of Classical Schizophrenia

**DOI:** 10.3389/fnhum.2022.818711

**Published:** 2022-03-03

**Authors:** Peter F. Liddle, Elizabeth B. Liddle

**Affiliations:** Centre for Translational Neuroimaging for Mental Health, School of Medicine, Institute of Mental Health, University of Nottingham, Nottingham, United Kingdom

**Keywords:** classical schizophrenia, disorganization, psychomotor poverty, negative symptoms, predictive coding, prediction error, polygenic risk score, inflammation

## Abstract

Current diagnostic criteria for schizophrenia place emphasis on delusions and hallucinations, whereas the classical descriptions of schizophrenia by Kraepelin and Bleuler emphasized disorganization and impoverishment of mental activity. Despite the availability of antipsychotic medication for treating delusions and hallucinations, many patients continue to experience persisting disability. Improving treatment requires a better understanding of the processes leading to persisting disability. We recently introduced the term classical schizophrenia to describe cases with disorganized and impoverished mental activity, cognitive impairment and predisposition to persisting disability. Recent evidence reveals that a polygenic score indicating risk for schizophrenia predicts severity of the features of classical schizophrenia: disorganization, and to a lesser extent, impoverishment of mental activity and cognitive impairment. Current understanding of brain function attributes a cardinal role to predictive coding: the process of generating models of the world that are successively updated in light of confirmation or contradiction by subsequent sensory information. It has been proposed that abnormalities of these predictive processes account for delusions and hallucinations. Here we examine the evidence provided by electrophysiology and fMRI indicating that imprecise predictive coding is the core pathological process in classical schizophrenia, accounting for disorganization, psychomotor poverty and cognitive impairment. Functional imaging reveals aberrant brain activity at network hubs engaged during encoding of predictions. We discuss the possibility that frequent prediction errors might promote excess release of the neurotransmitter, dopamine, thereby accounting for the occurrence of episodes of florid psychotic symptoms including delusions and hallucinations in classical schizophrenia. While the predictive coding hypotheses partially accounts for the time-course of classical schizophrenia, the overall body of evidence indicates that environmental factors also contribute. We discuss the evidence that chronic inflammation is a mechanism that might link diverse genetic and environmental etiological factors, and contribute to the proposed imprecision of predictive coding.

## Introduction

Schizophrenia remains an enigma. We know a large amount about causal factors. We also know a large amount about diverse pathological mechanisms, in both psychological and neuronal terms. We have had effective antipsychotic medications for more than 60 years. Despite all this, a substantial proportion of patients with schizophrenia still experience long term disability and shortened life expectancy.

A major issue is the heterogeneity of schizophrenia. Modern diagnostic criteria identify a spectrum of non-affective psychoses all characterized by a distorted perception of reality (American Psychiatric Association, 2013; World Health Organisation, 2018). Schizophrenia itself lies at the severe end, while at the mild end lies schizotypal personality disorder, in which the distortion of reality might not achieve psychotic intensity.

However, the heterogeneity is not simply degree of psychotic intensity. The presentation that we call schizophrenia is typically characterized by other features, some of which are associated with long term disability. This raises the question: is there a cluster of related psychological and/or neuronal processes that are associated with a tendency toward persisting disability, and which lie at the core of what we might call classical schizophrenia? Identifying such a core might open the door to therapies that lead to better long-term outcomes.

Liddle (2019) introduced the term classical schizophrenia to denote a disorder exhibiting not only the central features in the classical descriptions by Bleuler (1911) and Kraepelin (1919), but also characterized by pathophysiological processes that predispose to persisting disability. Liddle proposed disorganized mental activity, impoverished mental activity, and cognitive impairments as the three characteristic features of classical schizophrenia. The features of classical schizophrenia are summarized in Table 1.

**TABLE 1:** Clinical Features of classical schizophrenia (based on Liddle, 2019).

Core features	Persistent disorganization	Disorganized thought
		Disorganized affect
		Disorganized behavior

	Persistent impoverished mental activity	Poverty of speech
		Flat affect
		Diminished spontaneous movement

	Cognitive impairment	Slow speed of processing
		Impaired executive function
		Impaired working memory

Secondary features	Persistent impairment of role function	Impaired occupational function Impaired social function

	Reality distortion (typically episodic)	Delusions Hallucinations

Rathnaiah et al. (2020) demonstrated that the severity of classical schizophrenia can be estimated using symptom scores assessed using the Positive and Negative Symptom Scale (PANSS) (Kay et al., 1987); the Comprehensive Assessment of Symptoms and History (CASH) (Andreasen et al., 1992); or Symptoms and Signs of Psychotic Illness (SSPI) (Liddle et al., 2002).

Several lines of evidence support Liddle’s proposal. In a large non-clinical sample of young people, Dominguez et al. (2010) found that disorganization and negative symptoms predicted both subsequent overt psychosis and severity of functional impairment. Consistent with this, Ziermans et al. (2014) found greater disorganization in cases at high risk of schizophrenia, and that disorganization predicted poorer long term functional outcome. Perhaps most tellingly, Legge et al. (2021) found that genetic risk for schizophrenia was significantly correlated with disorganization (manifest as formal thought disorder and/or inappropriate affect), expressive negative symptoms (affective flattening and alogia), and impaired cognition, but not significantly with reality distortion. The finding of an association between polygenic risk score and disorganization is consistent with previous studies that have identified disorganization symptoms as the symptom cluster in schizophrenia with greatest heritability. However, the association between polygenic risk score for schizophrenia with not only disorganization but also with expressive negative symptoms and cognitive impairment suggests a heritable component for Liddle’s proposal for a cluster of traits constituting the core of a classical presentation of schizophrenia that is associated with long term disability.

To test the validity of this concept of a core of correlated features (disorganization, negative symptoms, cognitive impairments) associated with impaired role function, Rathnaiah et al. (2020) recruited a sample of patients in stable phase of illness, and used confirmatory factor analysis to verify a latent variable reflecting shared variance between these features. The verification of such a variable supports the proposition that these clinical features should be regarded as core features of classical schizophrenia. However it should be noted that the shared genetic origins do not necessarily account fully for the mutual relationships between these clinical features. As we shall discuss subsequently (in the section entitled Gene-Environment Interactions), it is likely that both genes and environment contribute to the clinical profile in an individual case.

In this paper we propose a plausible pathological mechanism at the core of classical schizophrenia in terms of an abnormality of predictive coding. Predictive coding refers to a range of mechanisms by which the brain generates internal models of the world that are successively updated in light of confirmation or contradiction by subsequent sensory information. Table 2 summarizes the predictive coding terminology employed in this manuscript.

**TABLE 2:** Terminology relevant to predictive coding.

Predictive coding	The mechanisms by which the brain makes predictions about the world, and successively updates them in light of further sensory information.
Forward model	The predictive model of the sensory consequences of an action. As the action is executed, the predicted sensory consequences are compared with actual sensory input. In the case of a mismatch, the motor command is updated, and a revised prediction generated.
Prediction error	The mismatch between predicted and actual brain-state. For instance, when the sensory consequences of an action are accurately predicted, the sensory input is down-weighted. Unpredicted sensory consequences constitute a “prediction error” and are likely to be more salient.
Salience of prediction errors	A prediction error means that the actual sensory input was unpredicted and therefore salient, in the sense of being more noticeable or “surprising.” The sensory input is also behaviorally salient as it indicates the need to adjust the internally generated prediction. A “salience network,” including insula and anterior cingulate cortex, plays a role in detecting and responding to salient information in general.
Teaching signal	The neural signal that indicates a prediction error and the need to adjust strategy and generate an updated prediction. The teaching signal is associated with release of dopamine from midbrain dopaminergic neurons, particularly when the error is in the value of a reward. It is likely that the teaching signal also produces enduring changes in expectations that guide prediction in similar circumstances in future.

Multiple lines of evidence indicate that predictive coding plays a cardinal role at multiple stages in the processing of information by the human brain, accounting for its efficiency. Instead of having to generate a fresh model with each volley of sensory of input, it needs only to match its predicted state against the state arising from relevant sensory input, and adjust for discrepancies. A discrepancy between the predictions and the sensory input represents a prediction error. If the prediction error exceeds the level expected from statistical noise, the predictive model of the world is updated. In Bayesian terms, the predictive prior is updated in light of the probability of the actual sensory input, given the prior likelihood, to provide an improved predictive model (posterior). In an influential account, Friston (2009) describes this process of minimizing surprise as the minimization of Free Energy (i.e., minimizing the amount correction likely to be required).

In the domain of perception, the predictive coding hypothesis provides an efficient mechanism by which an internally generated “feedforward” model requires only minor correction from “feedback” sensory confirmation (Rao and Ballard, 1999). What we perceive reflects our internally generated prediction after adjustment to minimize discrepancy with the sensory input, and explains why the world appears to stay still when we move our eyes, even though the image on the retina changes position.

More generally in the domain of motor control, we develop a forward model of the state of our brain and body as we execute an intended action (Wolpert and Ghahramani, 2000). Throughout execution, we compare our forward model with the incoming proprioceptive and external signals. We continuously adjust our action to minimize the discrepancy between prediction and sensory input. Thus, in this framework, the control of action is achieved via minimization of the discrepancy between endogenous prediction and sensory input. As the sensory consequences of our own actions are better predicted than the sensory consequences of an externally generated perturbation, this mechanism allows us to distinguish “self-caused” from “other-caused” sensory signals, and to discount the salience of the former—a mechanism postulated to explain “why you can’t tickle yourself” (Blakemore et al., 2000).

Predictive coding deficits have been invoked to account for delusions and hallucinations in psychotic disorders (Corlett et al., 2009). In the case of a motor act, if the proprioceptive and tactile feedback does not match the prediction generated by the motor command, the action might be perceived as alien. In more general terms, Adams et al. (2013) discuss the way in which imbalances between the precision of internally generated predictions and the weight allocated to precision of sensory evidence might account for both what they regard as “trait” phenomena of schizophrenia (which might include both disorganization and impoverishment of mental activity) and “state” phenomena (acute psychotic symptoms such as hallucinations). In particular they propose that trait abnormalities might arise from a decrease in the precision of internally generated predictions (or failure to down-weight sensory evidence), while acute psychotic symptoms might arise from a compensatory increase in the precision of internally generated predictions (or decrease in weight allocated to sensory information). They suggest that abnormality of glutamatergic or GABAergic transmission might play a cardinal role in trait abnormalities, while over activity of dopaminergic transmission might play a cardinal role in the acute psychotic state.

Brown and Kuperberg (2015) have reviewed the evidence that predictive coding deficits play a role in formal thought disorder, a key feature of disorganization. Sterzer et al. (2019) argue that schizophrenia involves a pervasive alteration in predictive coding at multiple hierarchical levels, including sensory and motor systems and also cognitive and value-based decision-making processes. They propose that impairments in various brain areas implicated in predictive coding account for the variety of psychotic experiences.

In this paper we examine the evidence that imprecision of internally generated predictions lies at the core of classical schizophrenia. Imprecise predictive models result in failure to down-weight what would otherwise be expected sensory stimuli, increasing their salience and the rate of error signals. Our hypothesis is similar to the proposal of Adams et al. (2013) that trait abnormalities might arise from a decrease in the precision of internally generated predictions (or failure to down-weight sensory evidence), but we propose that this “trait” reflects the “core” process in the pathway to classical schizophrenia: persistently imprecise predictions generate percepts that are both salient and tangential, reflected in disorganization symptoms, while a steady stream of minor error signals elevate net background dopamine levels and increase the risk of acute psychosis. Conversely, over-time, chronic errors may reduce the efficiency of decision-making, slowing cognition and action and giving rise to the psychomotor poverty and cognitive impairments of classical schizophrenia.

In accord with the observation that the genetic variants expressed in glutamatergic and GABAergic neurons contribute to the polygenic risk score for schizophrenia associated with the clinical features of classical schizophrenia (Legge et al., 2021), taken together with the proposal by Adams et al. (2013) that reduced synaptic gain arising from abnormality of glutamatergic or GABAergic transmission might play a cardinal role in trait abnormalities, we propose that classical schizophrenia arises from imprecise priors resulting from reduced synaptic gain in pyramidal neurons.

In this account we place emphasis on the role of imprecise predictions in disorganization of mental activity, and to a lesser extent, in impoverished mental activity. Nonetheless, we discuss the possible mechanism by which such a core process might account for the diverse aspects of schizophrenia, including the occurrence of at least transient episodes of reality distortion. We explore the way in which interaction between genetic prediction and environmental factors might account for the characteristic time-course of the illness.

## Evidence for Excessively Imprecise Predictions

To support our proposal, we will examine evidence derived from studies of five types of brain processes: Mismatch Negativity (MMN); oddball target detection; self-initiated action; perceptual organization, and Post-movement Beta Rebound (PMBR).

### Mismatch Negativity

MMN is an electrophysiological feature elicited by deviant acoustic stimuli delivered within a train of repetitive standard stimuli: The deviant stimuli elicit a greater negativity in the scalp potential compared to the standard stimuli, at approximately 200 ms after the presentation of the deviant stimulus. The MMN occurs even when there is no requirement to make a response (unlike the requirement in an “oddball” target detection task). In fact, the stimuli of interest are usually presented while the participant engages in some other activity such as watching a silent movie. Typically, the deviant stimuli differ from the standard stimuli in features such as such as pitch, duration, or intensity. MMN appears to reflect an automatic “change detection” process when an acoustic event violates expectation, even when it has no direct behavioral relevance.

Reduction of MMN amplitude is well established in schizophrenia. Interestingly, a meta-analysis by Erickson et al. (2016) of data acquired in over a hundred cases at various phases of illness indicated that MMN impairment appears to reflect vulnerability to disease progression in individuals at high risk of schizophrenia on the grounds of clinical features rather than a genetically determined risk for the condition. The association with vulnerability to disease progression suggests that the clinical features that predict severe illness may share a substrate with processes underlying reduced MMN, namely impaired detection of statistical irregularities in the environment.

#### Predictive Coding and Mismatch Negativity

Kirihara et al. (2020) reviewed the evidence indicating that reduced MMN amplitude might reflect altered predictive coding in schizophrenia. Studies using variants of the paradigm such as variation in the probability of the deviant stimuli and paradigms in which deviants entailed omission of expected stimuli, indicated that predictive coding is impaired in schizophrenia. For example, Baldeweg et al. (2004) measured MMN using a modified version of the “roving oddball” paradigm in which the last tone of each of a series of separate trains of auditory stimuli differs in duration from other stimuli. They found that healthy controls showed larger MMN amplitudes for oddballs following longer trains of repeated standards than for short trains, while MMN was not affected by the length of the train of repeated stimuli in patients with schizophrenia. These observations suggest less effective predictive coding in the patients. In light of inconsistent evidence regarding the role of familial factors underlying the MMN deficits in schizophrenia, we will return to the question of whether or not the MMN deficit is relevant to disease progression when we consider the role of gene-environment interactions in the cause of classical schizophrenia in the section entitled Gene-Environment Interactions.

#### Modeling Mismatch Negativity Deficits in Schizophrenia

The proposal that the MMN abnormality in schizophrenia reflects an abnormality of predictive coding is also supported by various mathematical models of the abnormality of MMN in schizophrenia. For example, Adams et al. (2021) developed a model of MMN employing Dynamic Causal Modeling. Their model comprised a distributed hierarchical network of brain regions implicated in generation of MMN: the inferior frontal gyrus, superior temporal gyrus and auditory cortex. They modeled the local circuits in the network nodes with inhibitory interneurons and spiny stellate cells interacting with superficial and deep pyramidal cells. The hierarchical model parameters were estimated according to Bayesian principles. The model successfully predicted a reduction in MMN in schizophrenia on the basis of local circuit parameters representing decreased synaptic gain in pyramidal cells in the patients. Synaptic gain of a neuron is the ratio of output signal to input. It can be adjusted by changes in synaptic strength mediated by glutamatergic and GABAergic transmission. The modeled decrease in synaptic gain represented increased self-inhibition of the pyramidal cells.

Using similar local circuit parameters, Adams et al. (2021) also applied Dynamic Causal Modeling to resting state EEG data, responses to steady state 40 Hz auditory stimulation, and resting state fMRI data in the same sample of patients. In all paradigms, the model was best fitted by local circuit parameters representing reduced synaptic gain in pyramidal neurons. In an analysis of relationships with symptoms, disinhibition in auditory areas predicted severity of positive symptoms.

Perhaps contrary to the evidence from meta-analysis (Erickson et al., 2016) indicating that MMN deficits in schizophrenia show no substantial familial influence, Adams et al. (2021) observed a decrease in MMN in first degree relatives of patients with schizophrenia.

### Oddball Target Detection

Oddball target detection tasks differ from MMN-eliciting tasks by requiring a motor response to the oddball stimulus. In typical oddball target detection paradigms, auditory tones at a particular frequency are presented randomly within a series of identical non-target stimuli, such has tones with a frequency different from the target. The participant is required to make a response, usually a button press, to target tones while refraining from responding to non-target tones. The oddball target stimuli elicit a characteristic series of event related potentials (ERPs), including a large positive-going deflection of the scalp potential, the P300, in a time window extending from 300 to 450 ms after the stimulus. The magnitude of the P300 is modulated by variation in target characteristics. Magnitude increases with decreasing target probability, suggesting, that like the MMN, P300 indexes the degree to which an internally generated expectation is violated.

A reduction in the magnitude of the P300 in schizophrenia is one of the best documented physiological abnormalities in schizophrenia. Many of the important clinical correlates of the P300 were described over two decades ago in Ford’s Presidential Address to the Society for Psychophysiological Research (Ford, 1999). Reduced P300 is both a state and a trait marker. In severe illness, the abnormality of the P300 is correlated with positive symptom score; it is also correlated with severity of persisting negative symptoms. Detectable abnormality persists even after symptom resolution. More recent studies confirm that deficits in P300 amplitude in both auditory and visual modalities emerge early in the course of illness, and precede onset of overt psychosis (Hamilton et al., 2019). P300 amplitude is also reduced in siblings of cases, suggesting a genetic contribution (Winterer et al., 2004; Groom et al., 2008).

The oddball target detection task entails perception, decision making and generation of motor responses, and thus might be expected to be a sensitive, though not specific, marker of abnormality in predictive coding. Oddball target detection tasks have only occasionally been addressed from the predictive coding perspective (e.g., Kolossa et al., 2012), at least in part because of difficulty distinguishing P300 modulations arising from mismatch in predictions regarding sensory stimuli, from mismatch related to the selection of response. Nonetheless, the theories of the role of predictive coding proposed by Friston (2009); Sterzer et al. (2019) and others imply that predictive coding plays a central role in the processes that contribute to perception and evaluation of the stimuli and/or planning the response in the oddball target detection task. As we shall discuss in the section entitled Brain Regions Engaged in Predictive Coding the brain regions engaged during predictive coding in healthy controls exhibit a marked overlap with the regions engaged during oddball target detection. In this section we will examine differences between patients and healthy controls in the effects of manipulations that modify endogenous (“top–down”) influence on the processing of information. Within a predictive coding framework, these manipulations would be expected to modify internally generated predictions. However, in the case of the fMRI data, we cannot distinguish effects arising from abnormality of the generation of predictions from differences attributable to abnormality of the response to prediction errors.

#### Reduced Signal to Noise Ratio

Precise predictive coding requires precise representation of both the content and timing of the neural representation of the coded information. An important parameter in modeling brain activity according to a predictive coding framework is the degree of confidence in the prediction (Adams et al., 2013). The ratio of signal-to noise in the neural representation of prediction would be expected to influence the confidence in the prediction. There is a substantial body of evidence regarding diminished cortical signal-to-noise ratio in patients with schizophrenia during the processing of information. Winterer et al. (2004) assessed background noise in frontal brain regions in discrete frequency bands across a range of frequencies extending from 0.5 to 45 Hz during auditory oddball processing. They quantified noise as the activity that did not exhibit a consistent temporal relationship to the presentation of the stimuli. They reported pronounced broadband cortical background noise over frontal cortex in patients with schizophrenia. A similar but less marked excess of noise was observed in clinically unaffected siblings. The frontal background noise predicted poor performance on frontal lobe cognitive tasks. There was a high intraclass correlation between sib-pairs suggesting high heritability of cortical background noise.

#### Inter-Trial Coherence and Phase Resetting

In a predictive coding framework, the brain needs not only to predict the causes of sensory input and the upstream neural consequences of that input but also when these events are likely to occur. Arnal and Giraud (2012) argue that slow endogenous cortical activity reflected in cortical delta (1–4 Hz) and theta band (4–8 Hz) oscillations play a role in predictive timing. In particular, delta band oscillations might play a role in the temporal organization of speech. In the context of selecting and responding to a target stimulus, transient bursts of low frequency oscillations might play a role in timing of neural events. The major features of the time-course of event-locked electrical potentials can be described as a superposition of transient delta and theta oscillations that are time-locked to the presentation of stimuli. The degree of phase locking is reflected in the consistency of the oscillatory phase across trial and can be quantified as inter-trail coherence (ITC) in the frequency band of interest. Consistency of phase of oscillations evoked by an event is achieved in part by consistent re-setting of the phase of ongoing cortical oscillations and partly by the addition of new oscillations with a phase that is locked to the event of interest (Martínez-Montes et al., 2008).

In a study of auditory oddball processing in schizophrenia, Doege et al. (2010) demonstrated that patients with schizophrenia exhibit significantly less ITC in the delta band and also significant less re-setting of the phase of ongoing delta oscillations, in comparison with healthy control participants. The resulting inconsistency of the phase of delta oscillations across trials contributed to the decrease in the observed magnitude of the P300. Furthermore the severity of the abnormality of phase resetting in the delta band was correlated with the severity of disorganization.

#### Processing of Speech Sounds During Oddball Target Detection

Healthy individuals exhibit increased neural activity in the left superior temporal gyrus in response to speech sounds compared to complex non-speech sounds. Ngan et al. (2003) employed fMRI to identify the pattern of brain activation associated with processing speech sounds in comparison with non-speech sounds in patients with schizophrenia, during an auditory oddball target-detection task in which the target stimuli were either speech sounds (such as “lif”) or non-speech sounds matched for acoustic complexity.

In comparison with healthy controls, patients with schizophrenia exhibited greater and more extensive activation for speech sounds in left superior frontal cortex, left temporo-parietal junction and right temporal cortex. The magnitude of the difference in activity in the left temporo-parietal junction was significantly correlated with severity of disorganization of speech. The finding of more extensive activation is consistent with the hypothesis that neural representation of speech sounds is less precise in patients. The more extensive activation of right temporal cortex suggests less clearly defined hemispheric lateralization of the processing of speech sounds, while the more extensive activation in the left temporo-parietal region suggests more widespread activation in brain regions normally engaged in processing speech sounds. The correlation of the aberrant activity in left temporo-parietal junction with severity of disorganization of speech suggests that disorganization of speech in schizophrenia is associated with failure to suppress inappropriate sounds related to speech.

#### Activity During Processing of Non-target Stimuli

Studies employing fMRI reveal widespread activation of the brain during the processing of target stimuli during the auditory oddball task (Kiehl et al., 2005). Furthermore, at least in well-established cases of schizophrenia, the activation during processing of target stimuli is diminished (Kiehl and Liddle, 2001). Performance of patients is usually impaired insofar as reaction times to targets are significantly longer in patients and there is a tendency toward more errors of omission in response to targets and errors of commission by failing to suppress response to non-targets (Kiehl and Liddle, 2001).

To minimize possible confounds in assessing brain activation arising from individual differences in task difficulty, Liddle et al. (2013) employed fMRI to assess brain activation during the processing of target stimuli and also the processing of non-target stimuli in an easy variant of the task in which the probability of targets was equal to that of non-targets, in early phase cases and also non-affected siblings of cases. Patients with schizophrenia and siblings showed significant hyper-activation to non-targets in brain areas activated by targets in all groups. The regions exhibiting hyperactivity included left superior frontal gyrus, fronto-insular cortex and bilateral temporo-parietal junction. In addition the patients and the siblings exhibited less deactivation to non-targets in Default Mode Network areas, including the precuneus, in which activity was suppressed during processing of targets in all groups.

These findings suggest that inefficient cerebral recruitment is a vulnerability marker for schizophrenia, made manifest by less suppression of activity in brain areas normally deactivated in response to task stimuli, and increased activation of areas normally activated in response to task stimuli. These findings are consistent with the hypothesis that vulnerability for schizophrenia is associated with inappropriate internally generated allocation of behavioral salience to non-target stimuli. With a predictive coding framework, this might be described as lack of precision in specification of prior expectation.

In a further test of the hypothesis that schizophrenia is associated with inappropriate internally generated allocation of behavioral salience to non-target stimuli, Liddle et al. (2016) employed Magnetoencephalography (MEG) to measure beta oscillations in the insula (a cardinal node of the salience network) during a relevance modulation task designed to compare activity during the processing of task-relevant stimuli with that during processing of task-irrelevant stimuli. The stimuli were images of either butterflies or ladybirds. The task-relevant stimulus type alternated between blocks. Beta oscillations were selected as the relevant measure on account of the evidence that beta oscillation mediate endogenously generated long range integrative signals (Fries, 2015). As predicted, healthy participants exhibited greater beta synchronization in the insula following processing of behaviorally relevant, as compared to irrelevant, stimuli. Patients with schizophrenia showed the reverse pattern: a greater beta synchronization during processing of irrelevant than relevant stimuli. Within a predictive coding framework, this might be described as inaccurate endogenous specification of expectation.

### Self-Generated Action

Using EEG, Ford et al. (2008) examined phase synchronization of brain oscillations in various frequency bands across trials (quantified as Phase Locking Factor, PLF) during self-paced button-pressing. Participants were required to press a button at will at time intervals of approximately 1–2 s. They argued that if PLF in sensorimotor cortex immediately preceding the motor action represents the “efference copy” of the action plan, it should be maximal in the hemisphere contralateral to the finger making the movement. Furthermore, if the role of the efference copy is to dampen the subsequent tactile sensory experience associated with the button press, the magnitude of the efference copy should related to the subsequent neural activity in sensori-motor cortex immediately after the button press.

They observed that in healthy controls, gamma band neural synchrony preceding the button press was maximal over the contra-lateral sensorimotor cortex, and was correlated with the amplitude of the somatosensory ERP evoked by the press. These effects were reduced in patients with schizophrenia. Furthermore, beta band neural synchrony preceding the button press was also reduced in patients. This reduction in beta synchrony was most marked in patients with avolition/apathy assessed using the Scale for the Assessment of Negative Symptoms (SANS).

It is noteworthy that volition/apathy scale employed in SANS contains items related to role function that reflect both disorganization and impoverishment of mental activity. In particular, in chronic illness, scores for poor grooming and hygiene, and for impersistence at school or work, are correlated more strongly with disorganization, whereas physical anergia is correlated more strongly with impoverished mental activity (Liddle, 1987).

### Perceptual Organization

Perceptual organization is the process of organizing sensory information into coherent patterns that represent objects, groups of objects and whole scenes, and our bodily relationship to them. Perceptual organization entails interpreting sensory input in light of internally generated predictions about relationships between percepts. Imprecise predictions would be expected to lead to impaired perceptual organization. There is extensive evidence for abnormalities of visual perceptual organization in schizophrenia. In a meta-analysis, Silverstein and Keane (2011) found that abnormalities of the organization of complex visual information are consistently found in schizophrenia, but are rare in other psychotic disorders such as bipolar mood disorder. Furthermore, in schizophrenia, these abnormalities are associated with severity of disorganization symptoms, and with poor premorbid functioning and poor prognosis. Thus, they are associated with features of classical schizophrenia. However, in light of evidence that disorders of perceptual organization are not prominent in the early phase of illness, Silverstein and Keane (2011) concluded that impairment of perceptual organization reflects illness progression.

In an fMRI study Silverstein et al. (2009) found that during a contour integration task, patients with schizophrenia exhibited diminished activation of visual association cortex during a perceptual organization task in comparison with healthy controls. Furthermore, patients with schizophrenia exhibited less activation compared with healthy controls in frontal, parietal and temporal regions during conditions in which integrated forms were perceived compared to random stimuli. In contrast the patients exhibited greater activation than controls in those brain areas during perception of random stimuli compared with integrated forms. These observations suggest a deficit in attentional enhancement of the perception of coherent forms relative to background information.

In a study of individuals with schizophrenia, first degree relatives of people with schizophrenia, individuals with bipolar disorder and healthy controls, Chen et al. (2005) found impairment of visual motion integration in the patients with schizophrenia, but not in the first-degree relatives, nor in patients with bipolar disorder, suggesting specificity to schizophrenia but indicating that it is not a familial trait.

In a structural and functional imaging study of schizophrenia and bipolar disorder, Palaniyappan and Liddle (2014) found that in comparison with bipolar disorder, schizophrenia was associated with increased functional connectivity between visual cortex and other regions of the brain during a working memory task, consistent with inadequate down-weighting of sensory evidence. Furthermore, Palaniyappan and Liddle (2014) found that the aberrant functional connectivity of the visual processing system predicted burden of persistent symptoms, consistent with the hypothesis that this abnormality in visual processing is specifically associated with classical schizophrenia.

### Post-movement Beta Rebound

Further evidence of imprecise prediction in schizophrenia is provided by abnormalities of the phenomenon of Post-movement Beta Rebound (PMBR). Beta synchrony decreases during the execution of a movement but then rebounds to a level higher than the baseline level over a period of several seconds following the movement. The magnitude of this rebound is influenced by the confidence in the motor plan. For example, Tan et al. (2014) assessed brain oscillations during a task in which healthy participants were required to move a joystick with the aim of moving a cursor toward a visual target presented on a computer screen. Unbeknownst to the participant, the relationship between the direction of movement the joystick and the motion of the cursor was manipulated during the task, creating uncertainty about the motor plan and inducing inaccurate responses. Tan et al. (2014) found that in the healthy participants, the magnitude of PMBR was lower when the error in direction of movement of the cursor was larger. In a subsequent study of healthy participants, Tan et al. (2016) employed Bayesian modeling to estimate the anticipated uncertainly in the environmental feedback and the uncertainty in the feedforward estimation. They concluded that magnitude of PMBR reflects the confidence in the internal feedforward estimation during sensorimotor integration based on updating of plans according to Bayesian principles. A high amplitude PMBR might be regarded as an index of confidence in the current motor plan, whereas a low amplitude PMBR might indicate the need for adaptive changes driven by the sensory feedback.

Several studies reveal that the magnitude of PMBR is decreased in schizophrenia. Robson et al. (2016) found that the decrease in magnitude of PMBR is correlated with the severity of persisting symptoms during a stable phase of illness. In a partially overlapping sample of cases, Rathnaiah et al. (2020) demonstrated that the reduction in PMBR was correlated with severity of classical schizophrenia quantified by a latent variable representing the shared variance between severity of disorganization, psychomotor poverty, cognitive impairment and impairment of role function, as discussed in the Introduction.

In a study using MEG to measure oscillatory activity following a simple finger abduction movement, in separate samples of early phase cases of schizophrenia (within 6 weeks of commencement of antipsychotic medication) and cases with well-established illness (of duration at least 10 years), Gascoyne et al. (2021) confirmed that PMBR was significantly reduced in both samples of cases relative to matched healthy controls. The reduction was greater in the well-established cases. In the well-established cases the magnitude of the reduction was correlated with severity of disorganization symptoms.

In a MEG study of oscillations following a finger abduction movement in a non-clinical sample, Hunt et al. (2018) found that magnitude of PMBR was inversely correlated with severity of schizotypal features. The greatest contribution to the relative diminution of PMBR came from schizotypal features reflecting disorganization of mental activity. Schizotypal features reflecting impoverishment of mental activity also made a lesser but nonetheless significant contribution to the PMBR deficit.

Although PMBR is usually quantified by averaging the beta power across a time window following movement, observation of data acquired in single trials reveals that the smooth peak of beta activity observed in trial averaged data actually represents the superposition of transient bursts of beta activity with duration of order 150 ms occurring with increased probability in the PMBR time window. In a study employing concurrent EEG and fMRI to assess brain activity during the n-back working memory task, Briley et al. (2021) confirmed that beta-burst rate following button press responses was diminished in schizophrenia, and also in psychotic bipolar disorder, compared with healthy controls. The reduction was less marked in the bipolar cases. Furthermore, the magnitude of the deficit was correlated with severity of disorganization. Multivariate analysis confirmed that shared variance between the features of classical schizophrenia: disorganization, psychomotor poverty and cognitive impairment, was significantly correlated with the reduction beta-burst rate. The findings in bipolar disorder suggest that at least some cases satisfying DSM IV criteria for bipolar disorder exhibit features characteristic of classical schizophrenia.

Furthermore, the analysis by Briley et al. (2021) of the BOLD signal assessed using fMRI revealed that beta-bursts are associated with the reactivation of a pattern of brain activity representing task-relevant content of working memory. In the context of the n-back task, in which the target stimuli were letters of the alphabet, the spatial distribution of BOLD signal associated with the occurrence of beta-bursts included areas implicated in the articulation of speech, processing of speech sounds and also sensorimotor areas engaged in the required motor response. In patients with schizophrenia or psychotic bipolar disorder, the BOLD activation was more extensive than in healthy controls, consistent with less precise representation of the content of working memory. Furthermore, the occurrence of beta-bursts was also associated with suppression of activity in brain regions expected to be engaged in the ongoing executive processing during the working memory blocks, implying that beta-bursts are associated not only with reactivation of the response-relevant brain activity but suppression of competing brain activity.

On the basis of study of beta-bursts in humans, primates and rodents, Sherman et al. (2016) proposed a neuronal model of beta-burst generation in which the characteristic time-course of a beta-burst is generated by coincident arrival of relatively broad peak of neural activity in middle layers of the cerebral cortex (via thalamo-cortical fibers) with the arrival of strong spike of activity in superficial layers of cortex. Such a model is potentially consistent with the hypothesis that beta-bursts play a role in the comparison of an intended action with the sensorimotor feedback from the action. A large amplitude beta-burst might confirm the timing and content of the planned response, while diminution of the amplitude of the beta-bursts, as seen in patients with schizophrenia, might indicate less precise prediction.

## Macroscopic Brain Changes

### Gray Matter Abnormalities in Schizophrenia

Many studies report widespread gray matter deficits in psychotic disorder. A mega-analysis of structural MRI studies employing Voxel Based Morphometry revealed deficits in schizophrenia that are most marked in insula and anterior cingulate cortex and also occur in association cortex sites implicated in executive function and attention, including lateral frontal cortex and parietal cortex (Gupta et al., 2015).

The gray matter reductions reported in mental disorders can reflect diminution of cortical thickness, surface area or gyrification. Diminished gyrification, especially in the insula, might be more specific to schizophrenia (Sheffield et al., 2021). Patterns of gyrification are largely determined during prenatal development, consistent with a developmental origin for classical schizophrenia. However, it should be noted that some evidence from longitudinal studies indicates that frontal gyrification can diminish during the early years of a schizophrenic illness (Palaniyappan et al., 2013). Evidence suggests that in schizophrenia, local gyrification in a region is related to density of long range connections with that region (White and Hilgetag, 2011).

Although reduction of local gyrification index in schizophrenia compared with healthy control participants has been reported in diverse brain regions, the most marked reductions occur in left insula and frontal operculum, left superior and middle frontal gyrus, temporo-parietal junction bilaterally and precuneus (Palaniyappan and Liddle, 2012). These regions contain major hubs in brain networks engaged in the regulation of attention (Seeley et al., 2007).

In the combined structural and functional imaging study comparing schizophrenia with bipolar disorder discussed in the section entitled Perceptual Organization, Palaniyappan and Liddle (2014) confirmed that in the contrast of patients with schizophrenia with healthy controls, the patients exhibited diminished gyrification in left insula, left superior frontal gyrus, regions in the vicinity of the tempo-parietal junction bilaterally, and left precuneus and posterior cingulate. They also conducted a conjunction analysis to identify brain regions exhibiting both diminished gyrification and increased functional connectivity in patients with schizophrenia relative to patients with bipolar disorder. They observed a conjunction of these effects (assessed at a lenient threshold) in left anterior insula, left middle temporal gyrus, left precuneus and posterior cingulate, and in visual processing areas in the lingual gyrus and calcarine fissure bilaterally.

### Brain Regions Engaged in Predictive Coding

Although it might be expected that brain regions engaged in perception of diverse sensory stimuli and in the planning of diverse actions might be engaged during predictive coding, fMRI studies suggest that predictive processing commonly engages certain brain regions. Ficco et al. (2021) performed a meta-analysis of 45 studies of paradigms involving prediction violation, and 39 studies of task entailing encoding of predictions. They employed Activation Likelihood Estimation (ALE) to identify sites of activity common to the different studies, and also a meta-analytic connectivity method (Seed-Voxel Correlations Consensus, SVC). The ALE analysis identified sites in left anterior insula and left inferior frontal gyrus that were active during prediction violation. The tasks that involved prediction encoding engaged a distinguishable set of sites including right insula, bilateral inferior parietal lobule, the cuneus and the right middle frontal gyrus. Nonetheless, the connectivity analysis demonstrated that the sites engaged in prediction violation were connected to the sites engaged in prediction encoding. The SVC analysis identified a large, bilateral predictive network, which containing many network nodes involved in task driven attention and execution, including insula and dorsal ACC; lateral frontal cortex and parietal cortex and also sites in the cerebellum.

Given that violations of prediction will have high salience, the involvement of the insula in detecting prediction violation is consistent with its role in the processing of salient stimuli (Seeley et al., 2007; Sridharan et al., 2008). It is noteworthy that left insula is the site of greatest gray matter reduction in schizophrenia revealed by mega-analysis of Voxel Based Morphometry data (Gupta et al., 2015), as well as the site of the most marked abnormalities of gyrification in schizophrenia (Palaniyappan and Liddle, 2012). Furthermore, in the stable phase of schizophrenia, disorganization is associated with diminished resting state cerebral blood flow in the left fronto-insular cortex, and also in temporo-parietal junction bilaterally (Liddle et al., 1992).

**FIGURE 1:**
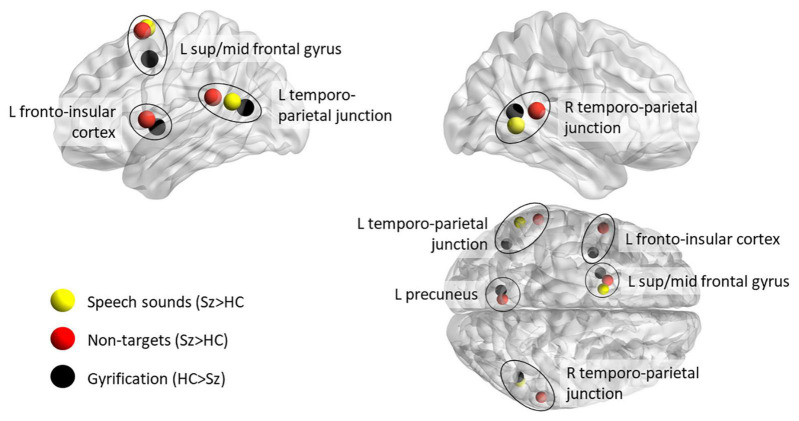
Comparison of regions of aberrant increase of brain activity in patients with schizophrenia during tasks likely to involve endogenous coding of predictions, with the regions in which local gyrification index is diminished in patients relative to healthy controls. Red spheres depict the loci of local maxima in clusters of voxels exhibiting greater activity in patients with schizophrenia relative to healthy controls during processing of non-target stimuli in a target detection task (Liddle et al., 2013); yellow spheres depict local maxima of aberrant activity in patients during processing of word sounds relative to non-word sounds (Ngan et al., 2003). The black spheres depict the local maxima in clusters of reduced local gyrification in schizophrenia compared with healthy controls (Palaniyappan and Liddle, 2012). Note that the clusters were irregular is shape and many extended beyond the sphere depicting the local maximum. In particular, the region of diminished gyrification with peak difference between patients and controls in the left middle frontal gyrus extended into superior frontal gyrus. The brain loci were visualized using Brain Net Viewer (Xia et al., 2013).

Figure 1 illustrates the similarity between the regions with most marked reduction of local gyrification in schizophrenia (Palaniyappan and Liddle, 2012) and the regions exhibiting an aberrant increase in activity in patients with schizophrenia relative to controls during processing of non-target stimuli in a target detection task (Liddle et al., 2013) and during processing of word sounds relative to non-word sounds (Ngan et al., 2003). It should be noted that the aberrant activity in the precuneus during processing of non-target stimuli is in a region of the Default Mode Network in which patients fail to exhibit the normal level of suppression of activity during processing of target stimuli. All of the regions depicted in Figure 1 in which patients exhibit aberrant activation lie within the network of sites engaged during predictive coding identified in the SVC analysis by Ficco et al. (2021), apart from the site in the precuneus, which Ficco et al. (2021) found to be anti-correlated with the predictive coding network.

## Molecular Brain Abnormalities

Modeling of EEG signals associated with Mismatch Negativity suggests that reduced synaptic gain arising from abnormality of glutamatergic or GABAergic transmission might play a cardinal role in aberrant predictive coding in schizophrenia (Adams et al., 2013, 2021).

Post mortem studies reveal consistent evidence of abnormalities of the morphology of dendrites of pyramidal (glutamatergic) neurons in the cerebral cortex, and of reduced levels of the protein synaptophysin, a sensitive marker for synaptic terminals, in frontal cortex and hippocampus, in schizophrenia (Hu et al., 2015; Osimo et al., 2019). *In vivo* measurements using Magnetic Resonance Spectroscopy (MRS) have been less consistent. Meta-analysis of MRS findings reveals decreased glutamate levels in frontal regions, especially anterior cingulate cortex, in both first episode psychosis and established schizophrenia (Sydnor and Roalf, 2020). However, a recent mega-analysis suggests that the reduction in glutamate might be attributable, at least in part, to antipsychotic medication (Merritt et al., 2021).

Post mortem studies also provide evidence for reduced transcription of genes implicated in GABA transmission in frontal cortex in schizophrenia (Hashimoto et al., 2008). *In vivo* measurements using MRS have been inconsistent but a recent meta-analysis indicates a significant reduction of GABA levels in anterior cingulate cortex, and a trend toward reduction in other frontal regions (Kumar et al., 2021).

## The Relationship Between Disorganization and Psychomotor Poverty

As discussed in the Introduction, Legge et al. (2021) found that the polygenic risk for schizophrenia is associated with both disorganization and with expressive negative symptoms. They also found a significant correlation between severity of disorganization and expressive negative symptoms in their cases. The expressive negative symptoms identified by Legge et al. (2021) (flat affect and alogia) are similar to the core negative symptoms reflecting impoverishment of mental activity, which Liddle (1987) labeled psychomotor poverty.

The findings of Legge et al. (2021) are consistent with the identification by McGorry et al. (1998) in a large sample of cases of First Episode Psychosis of a Bleulerian factor with contributions from symptoms reflecting disorganization and also symptoms reflecting psychomotor poverty. A similar factor with loadings on disorganization and psychomotor poverty symptoms was identified in another large study of first episode cases by Tonna et al. (2019). Furthermore these finding are consistent with the finding by Rathnaiah et al. (2020) of a latent variable with loadings on both disorganization and psychomotor poverty in stable cases of established schizophrenia (as referred to in the Introduction).

On the other hand, numerous other studies of the relationships between symptoms of schizophrenia report that disorganization and psychomotor poverty are distinguishable dimensions of psychopathology (Bilder et al., 1985; Liddle, 1987; Arndt et al., 1991). Thus, within an account of the pathological mechanism of classical schizophrenia it is necessary to propose a mechanism that accounts for the observation that disorganization and psychomotor poverty are related but nonetheless distinguishable clusters of symptoms.

Our proposal that imprecise predictive coding is a core feature of classical schizophrenia could account for the observed relationship between disorganization and psychomotor poverty, as both are potential consequences of imprecise prediction. Firstly, imprecise predictions will give rise to imprecise prediction errors and thus to erratic updating of predictions, leading, in turn, to poorly organized perception and action, manifest as disorganization symptoms. Secondly, if predictions are so imprecise that the prediction error is too diffuse to facilitate a coherently refined action plan, the process of generating action might be slowed or even fail to reach execution threshold at all, and manifest as impoverishment of motor activity. Furthermore, inconsistent updating of predictions over an extended period might result in a failure of consistent reinforcement of the pattern of neural activity coding the predictions, resulting in weakening of predictions and to diminished initiation of mental activity and behavior.

This possibility is supported by evidence regarding verbal fluency in schizophrenia. Allen et al. (1993) administered a verbal fluency test in which the person was required to generate as many words as possible within a given category in a 2 min period, on multiple occasions several days apart. They found that patients with schizophrenia do not suffer from diminished store of words. The variation between samples of words generated on different days suggested that cases with marked disorganization exhibit an inefficient search strategy, while cases with marked impoverishment of mental activity appear to terminate the search prematurely. This is consistent with the possibility that over an extended period of frequently occurring mismatches between predictions and experience, synapses in the networks engaged in generating specific predictions become weaker resulting in reduced ability to initiate a search for words.

## Contributions to Cognitive Impairment in Schizophrenia

In the study of polygenic risk scores and schizophrenia referred to in the Introduction, Legge et al. (2021) also examined the degree to which the polygenic risk for cognitive impairment contributes to clinical features of schizophrenia. Not surprisingly, they reported that the polygenic score for cognitive impairment occurring in diverse conditions contributes to cognitive impairment in schizophrenia. Thus the genetic contribution to cognitive impairment in schizophrenia arises from two distinguishable groups of genetic variants: the variants contributing to the polygenic risk for schizophrenia and from the variants contributing to polygenic risk for cognitive impairment in general. On further analysis Legge et al. (2021) found evidence that the polygenic risk score for schizophrenia contributes to cognitive decline rather than premorbid cognitive performance.

The evidence suggesting that the polygenic risk for schizophrenia predisposes to cognitive decline indicates that the expression of risk genes unfolds over time. It is plausible that disorganization is an early manifestation of the genetic risk for schizophrenia and that both psychomotor poverty and cognitive impairment develop over time as consequence of persisting lack of precision of predictions. This interpretation accounts for the evidence that the association of disorganization and psychomotor poverty with cognitive impairment is stronger in the chronic phase of schizophrenia than in the early phase. For example, Liddle and Morris (1991) found that in the stable phase of chronic schizophrenia disorganization is associated with impaired selection of mental activity (e.g., increased Stroop effect; unusual word choice during a verbal fluency test); whilst psychomotor poverty associated with impaired initiation of mental activity (e.g., decreased verbal fluency). Furthermore, this evidence for progression over time provides a pointer toward strategies for ameliorating at least some of the cognitive impairment of schizophrenia, and in turn, ameliorating impairment of role function that might arise from those impairments. Identification of the nature of the relevant neuronal processes offers the prospect of approaches to treatments that might prevent or reverse long term disabilities.

## Reality Distortion in Classical Schizophrenia

Reality distortion occurs in all psychotic illnesses, and is therefore not unique to schizophrenia. Nonetheless, reality distortion plays a major part in diagnostic criteria for schizophrenia employed in current clinical practice (American Psychiatric Association, 2013; World Health Organisation, 2018). The frequent occurrence of reality distortion in schizophrenia is unlikely to merely be an artifact of current diagnostic criteria. Simple schizophrenia, characterized by classical features without overt reality distortion and first identified by Diem in 1905, is relatively rare (Lally et al., 2019). Thus, an adequate description of classical schizophrenia must account for frequent occurrence of reality distortion in classical schizophrenia. In this regard it is noteworthy, as mentioned in the introduction, that in their longitudinal study of the emergence of psychotic illness in a large non-clinical sample of young people Dominguez et al. (2010) reported that prior disorganization and negative symptoms predicted onset of subsequent overt psychosis.

The hypothesis that the core aspect of classical schizophrenia is imprecise coding of predictions provides a possible clue to the mechanism by which the classical features of disorganization together with psychomotor poverty might precede the development of reality distortion and florid psychosis. Prediction errors are associated with the release of dopamine, which modulates the “teaching signal” responsible for updating of predictions (Schultz, 1998). Persisting imprecision of prediction would lead to frequent but unreliable prediction errors, leading to an increase in net dopamine (Jenkinson and Brown, 2011), increased dopaminergic transmission and amplification of unreliable teaching signals. In some circumstances, the strengthening of erroneous predictions might, lead either to delusional misinterpretation of exogenous signals or to misinterpretation of endogenously generated signals as exogenous signals (i.e., hallucinations). At times of stress, when levels of stress tend to magnify dopaminergic signals, classical schizophrenia might develop into florid psychosis.

## Time-Course of Classical Schizophrenia

The evidence regarding relationships between disorganization, psychomotor poverty and cognitive impairment, and also the relationships between these classical clinical features and reality distortion, indicates that the manifestations of classical schizophrenia evolve over time. It is therefore appropriate to summarize the time-course characteristic of classical schizophrenia.

### Prior to Illness Onset

Features of classical schizophrenia are discernible before illness onset in cases that subsequently develop schizophrenia. As mentioned in earlier, Dominguez et al. (2010) performed a 10 year follow-up study of a representative sample of over three thousand young people, recruited at age 14–24 years. Symptoms were assessed at three time points: at time 1, negative/disorganized score was based in scores for “indifference” and “thought incoherence”; at times 2 and 3; additional items measuring negative symptoms were also included. Negative/disorganized symptoms predicted positive symptoms over time. The co-occurrence of positive and negative/disorganized symptoms predicted functional impairment.

### Classical Features Occurring During the Prodrome

In many cases of schizophrenia, symptoms emerge gradually during a prodromal period. In one of the most comprehensive studies of the prodrome, Hafner et al. (1995) interviewed 232 cases in their first episode of overt schizophrenia, using an interview schedule designed to delineate the time-course and nature of the prodromal features. In that sample, the mean duration of the prodrome was 5 years. Non-specific symptoms such as restlessness, depression and anxiety were the most common symptoms. Nonetheless the ten most common features included features characteristic of classical schizophrenia: difficulty with thinking and concentration; lack of energy and slowness; poor work performance; and reduced interpersonal communication.

Several studies using computerized analysis of speech samples have demonstrated that subtle disorganization of speech in cases at risk predicts onset of overt psychosis and subsequent severity of negative symptoms (Bedi et al., 2015; Mota et al., 2017). As mentioned in the Introduction, Ziermans et al. (2014) performed a comprehensive assessment of symptoms and cognition in a sample of individuals identified as being at high risk of psychosis on the basis of clinical features. They found that severity of disorganization predicted poor functional outcome at 6 years (*r* = 0.55, *p* < 0.001).

In contrast, meta-analysis of the predictive power of duration of untreated psychosis (DUP) reveals that DUP has only very weak power to predict functional outcome. In a comprehensive meta-analysis, Penttilä et al. (2014) found a weak though statistically significant correlation between DUP and social function, but no significant correlation between DUP and occupational function.

### Long Term Evolution of Symptoms in Classical Cases

Pfohl and Winokur (1982) delineated the evolution of symptoms over a period of 35 years in a sample of hebephrenic/catatonic schizophrenia identified in a cohort of 500 cases admitted to mental hospital in a period 1935–1945, prior to the introduction of antipsychotic medication. At initial assessment, the hebephrenic/catatonic cases were characterized by disorganization of mental activity. The investigators examined case records reporting the presence or absence of 28 different symptoms assessed systematically on a yearly basis during the follow-up period. They found that symptoms reflecting reality distortion, such as hallucinations and delusions, had onset in the early phase of overt illness and in many cases, resolved within the first 5 years. In contrast, features characteristic of classical schizophrenia, such as loosening of associations, weakened volition, diminished social interaction and lack of engagement in productive activity were very prevalent in the early phases and usually persisted beyond 5 years. Furthermore, symptoms such incoherence of speech and flatness of affect became more prevalent in the longer term.

## Gene-Environment Interactions

As discussed in sections “The Relationship Between Disorganization and Psychomotor Poverty” and “Reality Distortion in Classical Schizophrenia,” life-long imprecision in predictive coding might in itself lead to an evolving course of illness. Imprecise predictive coding might initially produce disorganization and lead to psychomotor poverty, while predisposing to episodic reality distortion. However, such processes do not fully account for the characteristic time-course of classical schizophrenia. There is evidence that a diverse number of environmental factors might contribute to schizophrenia: maternal infection, obstetric complications, childhood abuse, brain injury, substance abuse (Gilmore, 2010).

Furthermore, the concept of Classical Schizophrenia embraces a specific clinical profile within the diverse clinical conditions that would be diagnosed as schizophrenia (or as a condition within the schizophrenia spectrum) according to either DSM-5 (American Psychiatric Association, 2013) or ICD-11 (World Health Organisation, 2018). It is likely that diverse environmental factors, and perhaps other genetic variants, contribute to the cause of psychotic conditions that do not exhibit the profile of classical schizophrenia.

Inflammation is a plausible process that might link diverse environmental factors to genetic factors contributing to schizophrenia. Watanabe et al. (2010) have reviewed the evidence suggesting that inflammatory processes mediated by cytokines such as epidermal growth factor (EGF) and interleukins provide a common pathway linking genetic and environmental factors. Genome wide association studies have revealed evidence of multiple genetic variants implicated in immune mechanisms, most notably variants of immunity-related genes located on chromosome 6 in the vicinity of the Major Histocompatibility Locus, are associated with schizophrenia (Shi et al., 2009).

Many of the environmental factors that predispose to schizophrenia involve stress on body tissues, including infection or metabolic processes that are associated with inflammation. Inflammatory cytokines, are elevated at onset of illness (Lesh et al., 2018) and through the course of the illness (Rodrigues-Amorim et al., 2018). It is noteworthy that in contrast to cytokine elevation observed in bipolar mood disorder, there is evidence that cytokine elevation observed at the onset of schizophrenia is associated with deficits in cerebral gray matter (Lesh et al., 2018). In light of the evidence for gray matter deficits in classical schizophrenia discussed in the section entitled Macroscopic Brain Changes, it is pertinent to ask whether or not inflammation might contribute to the clinical profile of classical schizophrenia.

Our hypothesis that the imprecision of predictive coding is a core process in classical schizophrenia raises the question: might inflammation impair predictive coding and in particular, be associated with electrophysiological abnormalities such as reduced MMN and PMBR? Emerging evidence supports this hypothesis. Jodo et al. (2019) demonstrated that neonatal exposure to the inflammatory cytokine, EGF, results in deficits of MMN in rats. In elderly men, persisting prostatitis and urinary tract infection is associated with cognitive deficits and with abnormality of MMN (Urios et al., 2019). Successful treatment with the phosphodiesterase-5 inhibitor, tadalafil, resulted in reduction of levels of the inflammatory cytokine, IL-6, recovery of the MMN to the level observed in healthy controls, and significant improvement in cognitive function (Urios et al., 2019). Investigation of the relationships between markers of inflammation and electrophysiological markers indicative of imprecise predictive coding, such as MMN and PMBR in schizophrenia is warranted.

## Discussion

In accord with our overarching goal of understanding of the processes that lead to poor functional outcome in schizophrenia, we have identified a specific clinical profile that we have designated “classical schizophrenia.” Classical schizophrenia is characterized by disorganized and impoverished mental activity that predispose to reality distortion, and is associated with risk of persisting impairment of role function. We have assembled evidence supporting the hypothesis that the core pathological process in classical schizophrenia is imprecise predictive coding.

This concept of classical schizophrenia is consistent with subtle but widespread abnormalities of brain structure, predominantly in association cortex. Some evidence indicates that abnormal gyrification, particularly in insula, lateral frontal cortex and superior temporal gyrus, is characteristic of classical schizophrenia. Evidence indicates that these regions play an important role in generating predictions and/or responding to prediction error.

While there is evidence that the genetic variants that contribute to the polygenic risk score for schizophrenia are especially predictive of the clinical profile of classical schizophrenia, it is likely that interaction between genes and environmental factors influence the time-course of classical schizophrenia. In particular, it is plausible that inflammation plays a cardinal role in mediating these interactions. Emerging evidence suggests that inflammation might contribute to the predictive coding deficits that we propose are at the core of classical schizophrenia. However, further evidence that inflammation might contribute to imprecise predictive coding is required. In particular, investigation of the relationship between inflammation and electrophysiological markers, such as MMN and PMBR, and also the relationships between inflammation and the proposed characteristic regional brain abnormalities revealed by structural and functional fMRI, is warranted.

In light of the association between polygenic risk score and the classical profile, it is noteworthy that Landi et al. (2021) demonstrated that polygenic risk score predicts poor outcome (quantified by measures such as course of disorder, classified as Continuous chronic illness/Continuous chronic illness with deterioration). However Landi et al. (2021) reported that the polygenic risk score does not add to the accuracy of prediction based on detailed analysis of electronic case records.

There is need for more sensitive measures of disorganization, both for the purpose of investigating mechanism and also for clinical prediction in early phase of illness. There is promising evidence that automated analysis of speech samples might help meet this need (see section entitled Classical Features Occurring During the Prodrome). In light of the observation that disorganization is also manifest as inappropriate affect, automated processing of facial expressions of emotion might also be helpful.

Furthermore, it would be of potential value to develop procedures for quantifying imprecise predictive coding from relevant measurements of brain function that might feasibly be employed in routine clinical practice. The evidence suggesting that PMBR is associated with predictive coding, together with the evidence that reduced PMBR is associated with clinical features of classical schizophrenia, raises the possibility that an assessment of beta oscillations using the clinically accessible technique of EEG, recorded during an appropriate attentional task, might have prognostic value.

One outstanding issue is whether or not classical schizophrenia is best regarded as a discrete category of psychotic illness, or alternatively, that the clinical profile of classical schizophrenia reflects a continuously distributed dimension of variation within the spectrum of psychotic illnesses. In light of the likelihood that a multiplicity of genes and environmental factors contribute to the cause of classical schizophrenia, there is likely to be a degree of heterogeneity within classical schizophrenia. A sharp categorical boundary is unlikely to exist. Nonetheless for the practical purposes of making a diagnosis and planning treatment, it would be useful to determine the degree to which classical schizophrenia can be identified as an illness that is categorically distinct from cases of psychotic illness that do not exhibit the classical profile.

In this paper we have discussed the evidence from studies of groups of cases, that the classical features reflecting disorganized or impoverished mental activity and cognitive impairment (listed in Table 1) predict long term disability. So far there is limited evidence that assessment of the relevant symptoms and cognitive measures are sufficiently sensitive and reliable to provide clinically useful estimates of prognosis in an individual case. However, the evidence that classical features are correlated with electrophysiological features such as inter-trial coherence during target detection tasks (Doege et al., 2010) and beta-bursts related to movement (Rathnaiah et al., 2020; Briley et al., 2021; Gascoyne et al., 2021) indicates a practical approach to enhancing prediction. It is practical to measure these electrophysiological features in routine clinical practice. It is plausible that a combination of measures of symptoms and cognition, together with such electrophysiological measurements would provide an estimate of risk of persisting disability that would facilitate planning treatment for an individual. The next step toward developing an effective predictive procedure would be identification of the optimal combination of these clinical and electrophysiological measurements at baseline for predicting role function in a longitudinal study of early phase cases over a period of a year or preferably longer.

We have also presented evidence from electrophysiological and brain imaging studies suggesting that the relevant pathological process entails imprecise predictive coding. Further investigation of the proposed pathophysiological mechanism might lead to development of improved treatment strategies. For example the modeling of the role of abnormal neural signaling in local circuits and also in long range connections in the electrophysiological features associated with classical schizophrenia, similar to the modeling performed by Adams et al. (2021), has the potential to lead to proposals for neuromodulation therapies effective in treating the features of classical schizophrenia.

## Data Availability Statement

The original contributions presented in the study are included in the article/supplementary material, further inquiries can be directed to the corresponding author/s.

## Author Contributions

PL proposed the concept of classical schizophrenia and drafted the manuscript. EL revised the manuscript. Both authors developed the hypotheses regarding the role of impaired predictive coding and approved the final draft.

## Conflict of Interest

The authors declare that the research was conducted in the absence of any commercial or financial relationships that could be construed as a potential conflict of interest.

## Publisher’s Note

All claims expressed in this article are solely those of the authors and do not necessarily represent those of their affiliated organizations, or those of the publisher, the editors and the reviewers. Any product that may be evaluated in this article, or claim that may be made by its manufacturer, is not guaranteed or endorsed by the publisher.
